# Disorders of compulsivity: a common bias towards learning habits

**DOI:** 10.1038/mp.2014.44

**Published:** 2014-05-20

**Authors:** V Voon, K Derbyshire, C Rück, M A Irvine, Y Worbe, J Enander, L R N Schreiber, C Gillan, N A Fineberg, B J Sahakian, T W Robbins, N A Harrison, J Wood, N D Daw, P Dayan, J E Grant, E T Bullmore

**Affiliations:** 1 Department of Psychiatry, University of Cambridge, Cambridge, UK; 2 Behavioural and Clinical Neuroscience Institute, University of Cambridge, Cambridge, UK; 3 Cambridgeshire and Peterborough NHS Foundation Trust, Cambridge, UK; 4 Department of Psychiatry, University of Minnesota, Minneapolis, MN, USA; 5 Department of Psychiatry and Behavioural Neuroscience, University of Chicago, Chicago, IL, USA; 6 Division of Psychiatry, Department of Clinical Neurosciences, Karolinska Institute, Stockholm, Sweden; 7 Department of Psychology, University of Cambridge, Cambridge, UK; 8 Department of Psychiatry, University of Hertfordshire, Hertfordshire, UK; 9 Clinical Imaging Sciences Centre, Brighton and Sussex Medical School, University of Sussex, Brighton, UK; 10 Center for Neural Science and Department of Psychology, New York University, New York, NY, USA; 11 Gatsby Computational Neuroscience Unit, University College London, London, UK; 12 NIHR Cambridge Biomedical Research Centre, Cambridge, UK

## Abstract

Why do we repeat choices that we know are bad for us? Decision making is characterized by the parallel engagement of two distinct systems, goal-directed and habitual, thought to arise from two computational learning mechanisms, model-based and model-free. The habitual system is a candidate source of pathological fixedness. Using a decision task that measures the contribution to learning of either mechanism, we show a bias towards model-free (habit) acquisition in disorders involving both natural (binge eating) and artificial (methamphetamine) rewards, and obsessive-compulsive disorder. This favoring of model-free learning may underlie the repetitive behaviors that ultimately dominate in these disorders. Further, we show that the habit formation bias is associated with lower gray matter volumes in caudate and medial orbitofrontal cortex. Our findings suggest that the dysfunction in a common neurocomputational mechanism may underlie diverse disorders involving compulsion.

## Introduction

One of the puzzling features of pathological behavior in disorders such as substance abuse—and even of more mundane daily choices such as consuming unhealthy foods—is that we repeatedly choose some behaviors despite knowledge that it has strongly negative consequences. A hypothesized explanation for this dissonance^1^ is that decisions can arise from two distinct, parallel systems of instrumental control, called goal-directed and habitual.^2,3^ In goal-directed control, we make choices that depend on their likely affective outcomes as predicted by a model of the environment. In habitual control, we make choices so as to repeat the actions that were previously rewarded. To put it in another way, goal-directed choices are prospective, whereas habitual choices are retrospective.^4^ Recent computational models have proposed that these two sorts of decisions arise from two different learning mechanisms, known as model-based and model-free reinforcement learning.^5^

The habitual system encodes choice tendencies divorced from their goals, leading to the suggestion that this system specifically may underlie the compulsive, repetitive action in disorders of addiction and compulsions.^1^ Normal behavior depends on the flexible integration of goal-directed and habitual control; if this breaks down in favor of just the latter, then pathology may ensue. Consistent with this idea, subjects with obsessive-compulsive disorder (OCD), characterized by repetitive thoughts and behaviors to avoid harm, exhibit signs of excessively habitual choices.^6^ Meanwhile, recent experimental work in healthy humans has introduced tasks that are able to tease apart the differential contribution of model-based and model-free learning mechanisms in acquiring new instrumental behaviors.^4^ Here we employ one such task to, first, examine whether these computational mechanisms are abnormally engaged in disorders of compulsivity, and, second, to identify neural substrates supporting both healthy and aberrant individual differences in these mechanisms.

**Figure 1:**
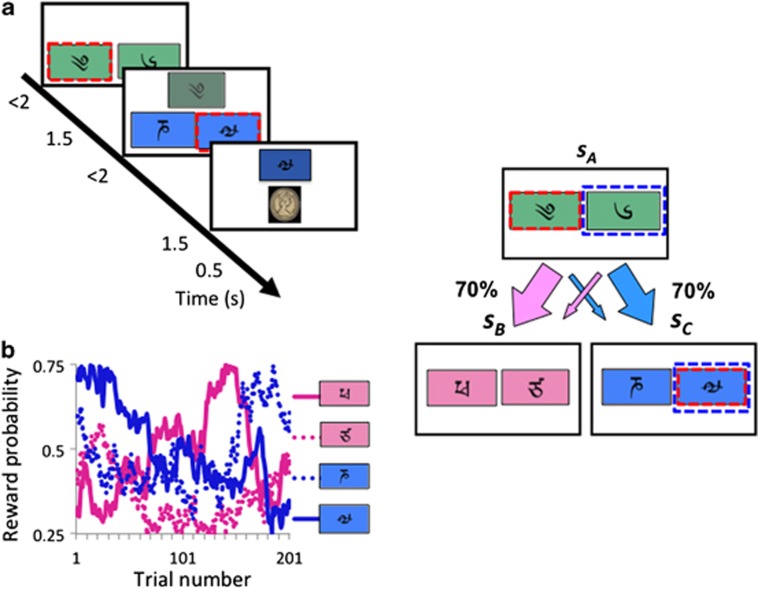
Sequential task. (**a**) Subjects chose between a stimulus-pair at the first stage leading with fixed probability to one of two stimuli-pairs in the second stage. Stimulus selection in the second stage leads probabilistically to a reward. (**b**) Example of reward probabilities for second-stage stimuli.

We employ a two-step sequential learning task used previously to show that healthy volunteers (HVs) simultaneously engage model-based and model-free learning processes.^4^ The task involves decision preferences that change on a trial-by-trial basis in a way that is expected to differ for the two sorts of learning, allowing their contributions to be distinguished. Subjects choose between pairs of stimuli at two stages (Figure 1). Each choice at stage 1 leads preferentially to a different stage-2 pair, according to a fixed probabilistic schedule (*P*=70%). Each individual choice at stage 2 leads to the chance of a reward which varies slowly between 25 and 75% over trials according to a random walk (Figure 1). Model-free learning uses the experience of the transitions and outcomes to calculate a reward prediction error which can reinforce the executed actions, giving rise to habitual behavior. By contrast, model-based learning builds a model of the state transitions, which it uses to produce goal-directed decisions by predicting the future prospects of each choice at stage 1 based on the updated values of the stimuli pairs at stage 2. The different characteristics of these two sorts of prediction and learning allow model-based and model-free adjustments to be teased apart.

In the first study here, we employed the two-step task to test subjects with disorders representing a broad range of compulsions, towards a natural or drug reward, or to avoid an aversive stimulus.^7^ These groups, comprising obese subjects with and without binge eating disorder (BED), abstinent psychostimulant (methamphetamine, Meth)-dependent subjects, abstinent alcohol dependent (EtOH) subjects and patients with OCD, are compared with matched HVs. We used the same monetary reward in the task which acts as a conditioned reinforcer across all behaviors to allow comparison between disorders. The relationship between obesity and substance use disorders has been of great interest but there is only limited evidence available in human studies.^8^ The rodent model of sucrose binge eating has many similarities with models of substance use disorders suggesting that this particular pattern of food intake, namely binge eating, may be a crucial subtype. We predicted that subjects with all compulsive disorders, BED, Meth, EtOH and OCD, would be associated with a shift away from model-based towards model-free control.

In the second study, we sought neural substrates supporting this variation both among HVs, and also in aberrant compulsion, here represented by BED. In particular, we assessed the relationship between model-free or model-based biases and gray matter volume specifically focusing on regions identified in previous studies including striatal regions, orbitofrontal, lateral prefrontal and parietal cortex.^4,9,10^ Lesion studies in rodents and imaging studies in humans have particularly pointed to ventromedial prefrontal and orbitofrontal cortices and dorsomedial striatum (dorsal caudate) as supporting goal-directed behaviors.^11,12^ Accordingly, we predicted that a bias towards habit formation would be associated with lower volumes in these regions. We further predicted that obese subjects with BED would similarly be associated with lower orbitofrontal and caudate volume relative to obese subjects without BED.

## Methods

The recruitment strategy has been reported previously^13^ and is described in Supplementary Materials. Subjects abstinent from Meth (1 week to 1 year abstinence) or EtOH (2 week to 1 year abstinence; at least 1 week off long-acting benzodiazepines), Obese subjects with and without BED and OCD subjects and their matched HVs were tested in the behavioral study. A subset of the obese subjects with and without BED returned for the imaging study. A different set of young HVs were recruited for the magnetic resonance imaging study. The study was approved by the University of Cambridge Research Ethics Committee and the University of Minnesota Institutional Review Board and informed consent obtained from all participants.

### Task

Subjects underwent extensive computer-based instructions explaining concepts and providing practice examples of changes in transition and probability, and the two-stage task structure.^4^ Instructions were self-paced and lasted 15 to 20 min. Subjects chose between a stimulus-pair at stage 1. The choice of a stimulus at stage 1 led with a fixed probability to one of two stimuli-pairs at stage 2 (*P*=0.70 or 0.30) with the other stimulus leading to the two stimuli-pairs with opposite probability (*P*=0.30 or 0.70). Choice of a stimulus at stage 2 led to a reward with probability varying slowly and independently over time (between *P*=0.25 to 0.75) (Figure 1). Four different reward probability distributions were used which was counterbalanced in each group. Subjects were given 2 s to make a decision at each stage. The transition between stage 1 to stage 2 was 1.5 s. The stimulus chosen in stage 1 remained on the screen in stage 2 as a reminder. The stimulus chosen in stage 2 remained on the screen in the feedback stage as a reminder. The outcomes were images of £1 in the United Kingdom and $1 USD in the United States. Subjects completed 201 trials divided into three sessions (7.5 s per trial, 8.38 min per session). The task was run using MATLAB 2011a.

### Computational model (adapted from Daw *et al.*)

The task has three states: the first-stage *state s*_*A*_, and two second-stage states *s*_*B*_ and *s*_*C*_; the two actions at each state are denoted *a*_*A*_ and *a*_*B*_.^4^

### Model-free temporal difference algorithm (habit)

The SARSA (λ) temporal difference (TD) algorithm was used to model the habitual strategy. Choices are based on the predicted long-run value (called *Q*_*TD*_ (*s*, *a*) of each action *a* at each state *s*, with the predictions being taught using the TD reward prediction error (δ Figure 1).

Each trial *t* includes a first-stage *state s*_1,*t*_ (=*s*_*A*_) in which an action *a*_1,*t*_ is chosen; this is followed by a second-stage state *s*_2,*t*_ (either *s*_*B*_ or *s*_*C*_) and action *a*_2,*t*_ and these by a reward *r*_2,*t*_ (=£1 or £0). A prediction error δ_*i,t*_ occurs following each stage *i* (=1,2) of each trial *t*: when the second-stage state is revealed, and at the terminal reward. Each of these updates the value *Q*_*TD*_ of the preceding state *s*_*i,t*_ and action *a*_*i,t*_:


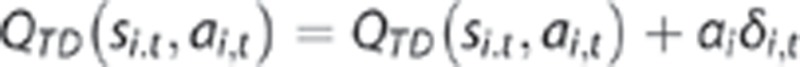


where


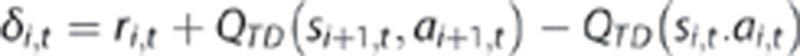


These expressions first update the stage-1 action value according to the value of the resulting stage-2 state, *Q*_*TD*_ (*s*_2,*t*_,*a*_2,*t*_) (with *r*_1,*t*_=0 as no reward is received at this stage). Next, the stage-2 value is updated in light of the reward *r*_2,*t*_; here the terminal value *Q*_*TD*_ (*s*_3,*t*_,*a*_3,*t*_) is defined as 0. A separate learning rate parameter is included for each stage’s update (α_1_, α_2_).

In addition to being updated by the stage-1 prediction error as described above, the first-stage action value is again updated according to the stage-2 prediction error at the conclusion of each trial, when the reward *r*_2,*t*_ is received. This update is added to the earlier one:


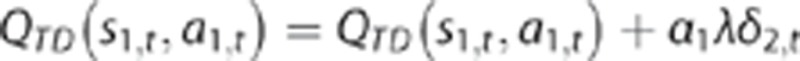


The extent of this update is determined by the eligibility trace parameter *λ*.

### Model-based reinforcement learning algorithm (goal-directed)

The model-based reinforcement learning algorithm calculated the stage-1 action value (*Q*_*MB*_) for each action, based on the probabilities that that action would lead to each stage-2 state (*P*(*s*_*B*_|*s*_*A*_,*a*_*A*_)=0.7; (*P*(*s*_*B*_|*s*_*A*_,*a*_*B*_)=0.3; and conversely for *s*_*C*_) and the values of those states. Thus, for each action *a*_*j*_ (*j*=*A*, *B*):


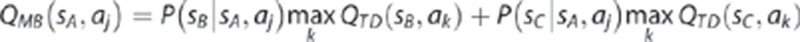


Here, the value of the stage-2 states is taken as the model-free value of the better action there, as model-free and model-based values coincide at the terminal state.

A net action value for each stage-1 action was then calculated, according to the weighted sum of the model-free and model-based values:


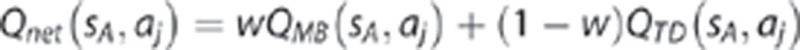


where *w* is the weighting parameter; *w*=0 indicates a reliance on model-free (habit) strategies and *w*=1 indicates a reliance on model-based (goal-directed) strategies. At the second stage, *Q*_*NET*_=*Q*_*TD*_.

Finally, the probability of a choice at each stage was calculated using the softmax equation in *Q*_*net*_:


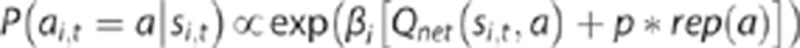


The inverse temperature parameter β_*i*_ is an index of choice reliability at each stage (β_1_, β_2_) with a lower value indicating greater choice randomness. The final parameter *p* controls perseveration (*P*>0) or switching (*P*<0) in the first-stage choices. Here, *rep*(*a*) is a binary indicator which is 1 if *a* is a stage-1 action and *a*=*a*_1_,_*t*-1_, and 0 otherwise.

The primary outcome of this analysis, *w*, along with other model parameters were compared between each patient group and their own HV using multivariate tests. Subject characteristics were compared using independent *t*-tests or Fishers Exact Test (Supplementary Tables S1 and S2). Parameters were tested for normality using Shapiro–Wilks test and square root transformation was used for *P*<0.05. Levene’s test was used to test for equality of variance. In Meth subjects, HIV+ and HIV− subjects and high nicotine (>1 pack per day) and low or no nicotine (<1 pack per day) were compared with multivariate analysis for model parameters. In the patient group, which had sufficient sample size (OCD) to compare between subjects on the same medication (antidepressants) and medication-free status, multivariate analysis was used to compare model parameters. The relationship between *w* and measures of severity for each disorder was assessed: BED (Binge Eating Scale), OCD (YBOCS), EtOH (AUDIT and duration of abstinence), Meth (duration of abstinence, duration of stimulant use and Penn Craving Scale) using Pearson correlation. Bonferroni correction applied for each disorder was used to assess significance. Other exploratory relationships between all model parameters and age, IQ, BDI (Beck Depression Inventory), UPPS Impulsive Behaviour Scale and gender using Pearson correlation and Chi square analysis. Matlab R2011A and SPSS 20 used for the modeling and statistics respectively.

### Behavioral outcomes

In the computational model, parameter values are determined by integrating effects associated with sequences of many choices. A more direct, though less powerful, way of assessing group differences is to examine pairs of successive choices, studying how any tendency of subjects to stay with the same stage-1 choice or switch following outcome (reward or no reward) depends on the frequency of the stage-1 to stage-2 transition (common (*P*=0.70) or rare (*P*=0.30)). Under the habitual system, a stage-1 choice would be more likely to be repeated (stay) when followed by reward, regardless of whether the transition was common or rare. Thus a habitual strategy would reflect a main effect of outcome in stay probability (Supplementary Table S3 and Supplementary Figure S1). Conversely, a goal-directed strategy would tend to switch its subsequent stage-1 choice if it was rewarded but the transition was rare. Given knowledge of the task structure, the other choice at stage-1 would more likely lead to the rewarded stage-2 choice. Thus, a goal-directed strategy would reflect an interaction between outcome × frequency.

We used a mixed effects logistic regression for the stay probability analysis with outcome (reward or no reward), frequency (rare or common), and group as factors, comparing all subject groups and HVs. Outcome, frequency, their interaction and the intercept were taken as random effects, that is, varying across subjects. We estimated the regression coefficients using the lme4 linear mixed effects package in the R statistical environment.

The imaging acquisition and analysis are described in Supplementary Materials.

## Results

Thirty-one obese subjects with BED and 31 without BED, 32 OCD, 23 abstinent Meth subjects and 30 abstinent EtOH subjects were compared with their own age- and gender-matched healthy HVs. Each group was matched with HVs in a 3:1 ratio. Thirty-three HVs were scanned for the first voxel-based morphometry study. Forty obese subjects with and without BED were scanned for the second voxel-based morphometry study. Subject characteristics are reported in Supplementary Materials.

We analyzed the data using a computational model of learning in which each individual’s trial-by-trial choices were fitted with the weighted combination of a model-free TD learning algorithm and a model-based algorithm with the weight being the primary parameter of interest.^4^ The best fitting model parameters were then assessed using multivariate analyses comparing each subject group with their own matched HVs.

**Figure 2:**
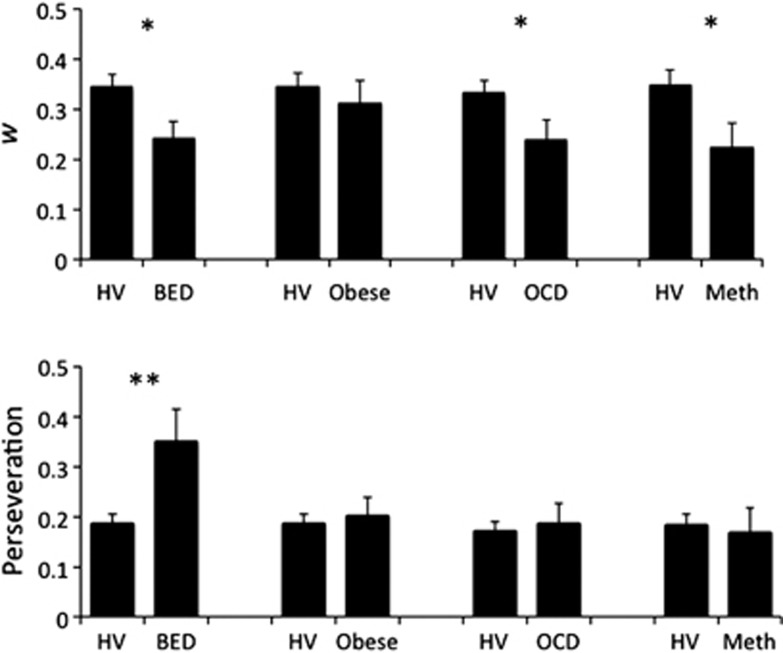
Computational algorithm parameters. (Top graph) Weighting parameter (*w*) and (bottom graph) perseveration indices. Patient group and matched healthy volunteer differences: *P<0.05 **P=0.001. BED, obese subjects with binge eating disorder; HV, healthy volunteer; Meth, methamphetamine-dependent; Obese, obese subjects without binge eating disorder; OCD, obsessive-compulsive disorder. Error bars represent s.e.m.

The weighting factor, *w*, provides an index of the relative engagement of a model-free (*w*=0) versus model-based (*w*=1) strategy. Obese subjects with BED, OCD and Meth subjects all had lower values of *w* compared with the matched HVs and thus were more likely to use habitual model-free processes (Figure 2, Table 1). Obese controls without BED and EtOH subjects did not differ from HVs. BED subjects also had lower *w* (F(3,58)=4.167, *P*=0.046) and higher perseveration scores (F(3,58)=4.406, *P*=0.040) compared with obese subjects without BED when covaried for age and gender.

In the BED subjects, higher Binge Eating Scale scores were negatively correlated with *w* (*R*^2^=0.18, *P*<0.05). In the EtOH subjects, weeks abstinent were positively correlated with *w* (*R*^2^=0.23, *P*=0.008). There were no significant differences between model parameters in OCD subjects on antidepressants (*N*=19) compared with those not on medications (*P*>0.05).

In the additional model parameters, there were only a few other differences noted between groups and their HVs. A secondary index quantifying perseveration at stage 1 (that is, the tendency to select the same stage-1 stimulus irrespective of reward outcome), demonstrated that BED subjects were more perseverative compared with HV. Other groups did not differ significantly from HV according to this measure. Also, in Meth-dependent subjects, we found greater choice randomness in stage 2 (lower inverse temperature parameter β_2_).

These results demonstrate that diverse disorders of compulsivity are accompanied by an excessive tendency toward model-free learning. We also conducted a parallel analysis of the behavioral data seeking markers of model-free and model-based learning more directly in subjects’ raw switching behavior, thereby relaxing some of the assumptions of the full computational model and visualizing the effects more directly.^4^ The results of this analysis (Supplementary Figure S1,Supplementary Table S3 and Supplementary Text) were substantially similar to the computational analysis.

**Figure 3:**
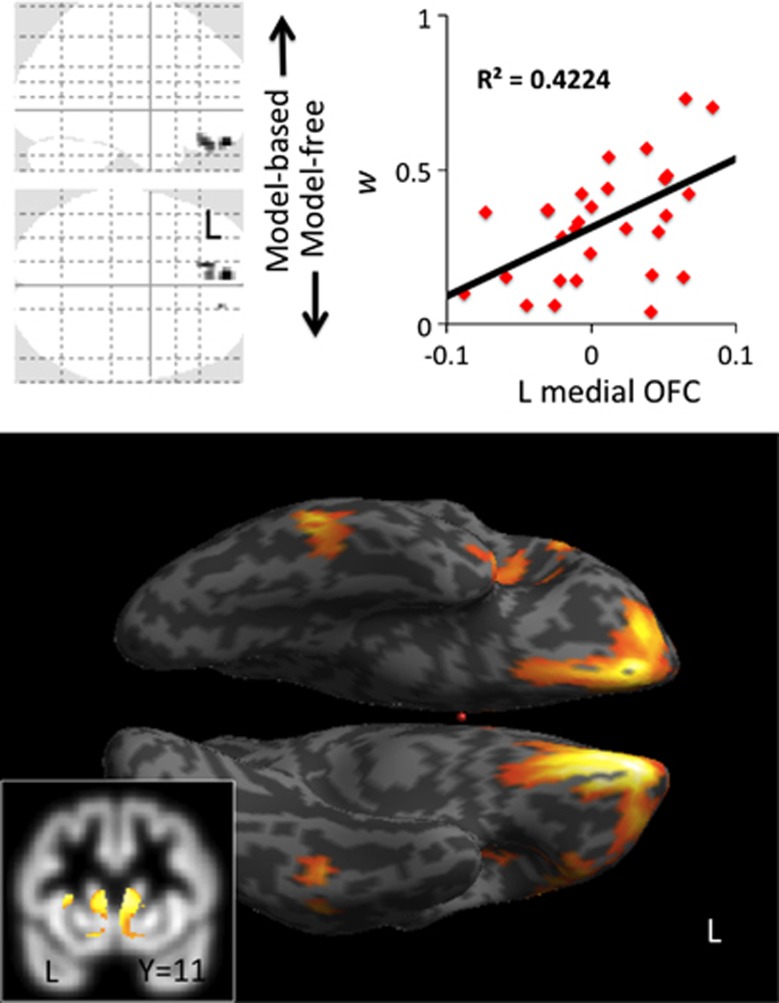
Voxel-based morphometry in healthy volunteers. The glass brain, inflated surface render and graph show a regression analysis with brain volume and *w*. The glass brain and surface brain are shown at FWE corrected *P*<0.05 and whole brain uncorrected *P*<0.005, respectively. The inset shows the same regression analysis with a striatal mask overlaid on the mean group T1 gray matter image. FWE, family-wise error; OFC, orbitofrontal cortical.

Next, we used voxel-based morphometry in a different set of HVs (mean age 23.22 (s.d. 2.75); 19 males) to examine how brain volume related to the relative engagement of model-based learning, as measured by the parameter *w*. Taken as an independent regressor, in HVs, *w* was positively correlated with left medial orbitofrontal cortical (OFC) volume (peak coordinates reported in Montreal Neurological Institute *x* *y* *z* (mm)=−8 54 −23, *Z*=4.90, cluster size=69, 87, 10 for three clusters, whole brain family-wise error (FWE) corrected *P*=0.01), with a positive direction of effect meaning that greater cortical volume was associated with a stronger tendency toward model-based learning (Figure 3). Conducting small volume correction (SVC) analyses for hypothesized regions in striatum, prefrontal and parietal areas, we found that *w* was also positively correlated with bilateral caudate volume (left: −9 5 6, *Z*=3.18, SVC corrected *P*<0.05; right: 8 6 8, *Z*=3.45, SVC FWE corrected *P*<0.05; Figure 3) but not putamen or ventral striatum. Furthermore, *w* was also positively correlated with bilateral lateral prefrontal (Brodmann area 46, right: 53 23 26, *Z*=4.11, SVC FWE corrected *P*=0.01; left: −45 20 27, *Z*=3.94, SVC corrected *P*=0.02) but not parietal cortex volume. The perseveration index was not associated with any significant correlations. The inclusion of age and BDI scores as covariates of no interest in a subanalysis did not change the findings.

**Figure 4:**
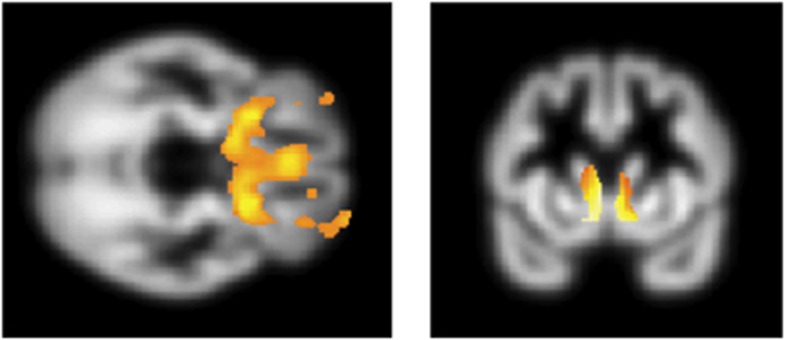
Voxel-based morphometry. Contrast of obese subjects with binge eating disorder−obese subjects without binge eating disorder shown at whole brain uncorrected *P*<0.005 and with caudate mask. Volumetric findings are overlaid on the mean group T1 gray matter image.

Finally, we examined whether these same neural systems were associated with pathological compulsive disorders, where excessive model-free behavior had been observed. We compared 20 obese subjects with and 20 without BED focusing on the regions shown to be associated with normal variation in model-free versus model-based learning in our HV study (medial OFC, caudate and lateral prefrontal cortex). Obese subjects with BED had lower left ventral striatal volume (−20 15 −9, *Z*=4.91, cluster size=9, FWE whole brain corrected *P*<0.05) and left lateral OFC volumes (−32 47 −11, *Z*=4.77, cluster size=5, FWE whole brain corrected *P*<0.05) compared with those without BED. Obese subjects with BED also had lower bilateral medial OFC volume (3 36 −17, *Z*=3.63, SVC FWE corrected *P*<0.05) and bilateral caudate volume (left: −9 17 −15, *Z*=3.68, SVC FWE corrected *P*<0.05 and right: 9 14 −12, *Z*=3.42, SVC FWE corrected *P*<0.05) compared with those without BED (Figure 4). With the addition of the model-based parameter *w* from behavior as a covariate, these group-wise medial OFC, caudate and ventral striatal findings were no longer significant (no voxels observed in these regions including when lowering the threshold to an uncorrected *P*<0.05) suggesting that the individual differences in learning bias might largely explain differences in cortical and striatal volumes between groups of obese subjects with and without BED.

## Discussion

A wealth of preclinical studies and influential theory suggest that stimulant addiction is associated with abnormal habit expression;^1^ similar suggestions have been made for repetitive avoidance behaviors (OCD). Here, we show that these disorders are also associated with a significant shift in habit formation, evident early in the learning of a new decision problem, and that the abnormality can be quantified in terms of a detailed computational learning mechanism with strong neural foundations, model-free learning.^3,5^ Although abstinent EtOH subjects did not differ from HVs, this lack of a difference may be in part mediated by abstinence. Early abstinence was associated with greater habit formation with a shift towards greater goal-directed behaviors with prolonged abstinence. This relationship suggests a possible role for top-down volitional control in decreasing habit formation. We similarly demonstrate greater model-free habit formation in obese subjects with binge eating behaviors, as compared with those without, suggesting that this neurocomputational mechanism may be commonly implicated across a broad range of disorders and in particular supporting similarities between the subtype of binge eating and substance use disorders.

Our results also implicate defined neural substrates in these effects. We show that in HVs, lower gray matter volumes in the caudate, medial OFC and lateral prefrontal cortices were associated with a greater shift towards model-free habit formation. These findings dovetail with rodent lesion and human imaging studies implicating these regions in model-based goal-directed behaviors. Blood-oxygen-level dependent activity in various cortical regions covaries with aspects of model-based learning in HVs: for instance, the state prediction error, or the discrepancy between the observed and expected state transition is represented in the lateral prefrontal cortex and intraparietal sulcus.^9^ Further evidence of the role of cortical regions comes from rodent studies showing that their ability to solve (Pavlovian) reversal tasks when the identity rather than the value of outcomes changes depends on the orbitofrontal cortex—this is another sign of model-based rather than model-free processing^10,14^ although these regions may be more likely lateral rather than medial orbitofrontal cortex. Using a three-step decision tree task of which this current task is its predecessor, model-based and model-free values were shown to be encoded in the caudate and putamen, respectively, whereas the ventromedial prefrontal cortex accessed both systems.^15^ The present results also tie these systems to compulsion, in that BED is similarly associated with lower bilateral caudate and bilateral medial OFC and left ventral striatal gray matter volumes, though not with lateral prefrontal volume. These volumetric differences between obese subjects with and without BED have not been reported in previous studies. Our findings suggest that habit formation related to binge eating may be mediated by a medial OFC—caudate network, which may also mediate variation in these functions among HVs.

The neural areas where we see structural differences related to model-based learning coincide well with areas implicated in goal-directed behavior in rodent lesion and human imaging studies. From studies in rodents, we know that lesions of the posterior dorsomedial striatum or prelimbic cortex prevent the expression of goal-directed learning instead leaving inflexible habitual choices that are insensitive to contingency degradation and outcome devaluation.^11,16^ Similarly, human functional imaging studies focusing on the encoding of reward value signals relevant for action selection implicate the medial orbitofrontal cortex extending dorsally along the medial prefrontal cortex. These regions represent action–outcome associations^17^ separate from stimulus-related value signals.^18^ The caudate is also implicated in the online computation of action–outcome contingency to guide goal-directed learning.^19,20^ Our findings are convergent with these observations, suggesting that greater habit formation may be related to impaired representation of action–outcome associations during goal-directed learning with lower caudate and medial OFC volumes.

Abnormal orbitofrontal and caudate gray matter volume have also been demonstrated in substance use disorder subjects. Abstinent stimulant use disorder subjects have decreased medial orbitofrontal cortex volumes,^21^ with lower volumes associated with impaired decisions in a modified gambling task.^22^ Meth dependence is similarly associated with decreased orbitofrontal cortex volume^23^ and may be related to comorbid nicotine use.^24^ Meth dependence is also associated with lower striatal D2 receptor availability correlating with lower metabolism in the OFC.^25^ Furthermore, active Meth use or early abstinence is associated with lower caudate volumes independent of comorbid nicotine use,^24^ with increases in volume with prolonged abstinence.^26^ Thus, greater habit formation in Meth dependence may be mediated by orbitofrontal cortex and caudate abnormalities, similar to our results in BED here.

Our data are also consistent with marked overlaps in addiction towards drug and food rewards. In rodents, sugar bingeing demonstrates addictive-like properties including enhanced responding for sugar after abstinence, amphetamine cross-sensitization and nucleus accumbens dopamine release.^7^ Although evidence in the preclinical literature points towards such similarities, evidence to support this link in the human literature is more limited.^8^ In humans, food stimulation in BED subjects is associated with greater striatal dopamine release.^27^ Similar to psychostimulant dependence, studies in obese humans demonstrate lower striatal dopamine D2 receptor binding along with lower prefrontal metabolism including the medial OFC.^28^ Body mass index is also associated with lower OFC volume in older females.^29^ Our findings provide convergent data, demonstrating similar abnormalities in habit formation in stimulant addiction and binge eating, a specific subtype of obesity, possibly mediated by caudate or medial OFC impairments.

We compared these groups with patients suffering from OCD, a seemingly quite different case of compulsion as it is based on avoidance rather than appetitive motivation. Individuals with OCD have demonstrated impairments in implicit action–outcome representation, as reflected in ‘slips of action’ to previously rewarded stimuli despite negative outcomes, and impaired explicit recall of action–outcome associations.^6^ OCD is also associated with greater habitual avoidance choices following overtraining with a shock avoidance task and outcome devaluation.^30^ Decreased OFC volume is commonly observed in OCD in the region-of-interest-based analyses.^31^ However, perhaps partly because of methodological issues, a recent meta-analysis of volumetric studies in OCD failed to confirm that OFC volumes were abnormal although it did show significantly enhanced caudate volumes.^32^ Impaired functional connectivity of the OFC has been demonstrated in both OCD and stimulant dependence suggesting overlapping OFC functional abnormalities that may link with habit formation.^33^

Anatomical studies of primates and humans have shown that the medial OFC projects to the ventral striatum and ventromedial caudate, and the dorsolateral prefrontal cortex projects to the dorsal caudate.^34,35^ Although we showed a relationship between *w* and medial OFC and caudate, we did not show a significant relationship with ventral striatum. Thus, whether these represent engagement of two different fronto–subcortical pathways in HVs is not clear. We showed that obese BED have lower volumes in bilateral medial OFC and caudate and left ventral striatum.

In addition to these shifts in habit formation across groups, we further show differences between disorders, which may help explain the differences in clinical presentations. BED subjects perseverated more in their stage-1 choices irrespective of the outcome, a measure independent of the habit formation index. Thus, binge eating is characterized by both outcome-dependent habitual choices as a function of previously rewarded actions, and also by greater perseveration irrespective of outcome, suggesting generalized impairments in cognitive flexibility.

In Meth-dependent subjects, in addition to enhanced habit formation, we also found greater choice randomness in stage 2 (lower β_2_). In reinforcement learning models, decreasing β is exactly equivalent to decreasing the effective magnitude of the reinforcement outcome (as they always appear multiplied together). These findings are thus consistent with reports of decreased sensitivity to monetary rewards in psychostimulant-dependent subjects including decreased subjective discrimination of monetary reward gradients,^36^ and decreased reaction times and lateral orbitofrontal responsivity to monetary reward outcomes.^37^ However, importantly, across all the patient groups the differences in choice at the first level (where model-based and model-free learning are distinguished) were related specifically to *w*, which indexes the relative engagement of these processes. No differences were noted in the first-stage inverse temperature parameter β_1_, which characterize the overall reliability of that behavior given the modeled quantities. These results suggest that the group differences were not secondary to overall sloppier performance or engagement in the task (which would all be expected to translate into lower β_1_) but instead to the more specific nature of subjects’ learning strategies. Pathological changes secondary to the drug use such as reactive microgliosis have been shown in Meth-dependent subjects in regions including the orbitofrontal cortex, striatum and midbrain.^38^ These observations highlight the differential consequences of drug and food use and may underlie our observed behavioral differences and potential changes with abstinence.

Although we show a relative shift from goal-directed to habit formation and emphasize the role of habit formation, whether this is related to a decrease in goal-directed or an increase in habit formation remains to be established. The effects may be related to a decrease in goal-directed behavior, which would also be consistent with the medial OFC and caudate findings. Equally, goal-directed learning is more cognitively demanding, and so places greater demands on resources. Even in HVs, the simultaneous performance of a demanding task results in a shift towards habitual behaviors^39^ and this may be exacerbated in patients suffering from various psychiatric conditions. Similarly, stress, which is relevant in disorders of addiction, has been associated with a decrease in medial prefrontal and caudate volumes, along with a shift towards greater habits.^40^ Further studies would be necessary to disentangle these effects.

There were several limitations to the study. As this is a cross-sectional study, whether the abnormalities are state-specific and secondary to the drug or behavior or trait-specific and predispose towards the addiction process is not known. All subject groups had higher depression scores relative to HV. Similarly, Meth-dependent,^41^ BED^42^ and OCD^43^ subjects are commonly associated with depression. Notably, we show that depression scores are unrelated to these measures suggesting these factors to be relatively unimportant. We show a similar direction of effect across several groups but do not show an increase in goal-directed behaviors. An increase in goal-directed behaviors has been previously shown in response to Levodopa^44^ suggesting that our findings are related to group differences rather than task insensitivity. We also use a common conditioned reinforcer of money across groups to allow for comparisons. Further studies using group-specific incentive reinforcers are indicated. Although we did not show any difference between OCD subjects taking antidepressants or those who were medication-free, this may be related to sample size issues, so further studies would be desirable.

The shared patterns of abnormalities we report here suggest that abnormal habit formation via model-free learning may be an underlying neurocomputational mechanism, associated with abnormal caudate and medial OFC volume, which contributes to a dimension of compulsivity common to these disorders. The influence of abstinence in EtOH subjects highlights a possible role for drug exposure in the transition towards habit formation. Our findings dovetail with the current trend in defining mechanistically based dimensional rather than categorical approaches to psychiatric classifications.^45^ Similarities in habit formation highlight overlaps between the subtype of binge eating in obesity and substance use disorders, explaining in part the pathological choice towards high-calorie food consumption despite negative consequences. Crucially, we also identify differences between disorders, which might underlie the differences in clinical presentation and the differences between the consequences of drug and food. Cognitive or pharmacological strategies^46^ to shift the bias away from habit formation towards forward planning model-based goal-directed approaches may be therapeutically useful.

## Figures and Tables

**Table 1:** Inferred parameters

	N	w	P	*β*_*1*_	*β*_*2*_	*α*_*1*_	*α*_*2*_	λ	*LL*
HV	93	0.343 (0.239)	0.187 (0.177)	4.892 (4.002)	3.124 (1.935)	0.416 (0.309)	0.403 (0.276)	0.579 (0.289)	214.724 (41.704)
									
BED	31	0.242 (0.183)	0.349 (0.360)	3.578 (3.250)	3.435 (2.820)	0.469 (0.318)	0.338 (0.273)	0.588 (0.350)	222.961 (42.450)
									
F *P*		4.5620.035	12.264 0.001	2.535 0.144	0.395 0.531	0.540 0.464	1.245 0.267	0.007 0.935	0.981 0.324
									
HV	93	0.345 (0.242)	0.186 (0.175)	4.883 (3.980)	3.200 (1.944)	0.416 (0.305)	0.400 (0.281)	0.585 (0.287)	214.432 (41.077)
									
Obese	31	0.312 (0.239)	0.203 (0.191)	5.573 (3.939)	2.961 (1.959)	0.435 (0.342)	0.384 (0.295)	0.678 (0.280)	213.165 (48.127)
									
F *P*		0.448 0.504	0.173 0.678	0.780 0.379	0.457 0.500	0.062 0.803	0.069 0.793	2.351 0.128	0.020 0.887
									
HV	96	0.333 (0.243)	0.173 (0.173)	4.901 (3.922)	3.196 (2.028)	0.411 (0.304)	0.398 (0.276)	0.574 (0.290)	214.496 (41.227)
									
OCD	32	0.239 (0.211)	0.188 (0.211)	4.937 (4.271)	2.913 (1.780)	0.398 (0.276)	0.380 (0.306)	0.502 (0.332)	217.499 (48.610)
									
F *P*		4.133 0.044	0.009 0.925	0.002 0.964	0.537 0.465	<0.001 0.992	0.110 0.741	1.493 0.224	0.092 0.763
									
HV	66	0.347 (0.239)	0.185 (0.172)	4.806 (3.159)	3.323 (1.768)	0.445 (0.283)	0.417 (0.226)	0.574 (0.317)	215.055 (43.921)
									
Meth	22	0.224 (0.218)	0.168 (0.236)	4.667 (3.902)	2.283 (1.598)	0.3144 (0.293)	0.336 (0.322)	0.480 (0.310)	230.451 (45.148)
									
F *P*		5.713 0.029	0.149 0.704	0.029 0.856	6.188 0.015	3.583 0.062	1.700 0.196	0.145 0.704	1.928 0.168
									
HV	90	0.331 (0.237)	0.184 (0.179)	5.131 (4.039)	3.235 (2.073)	0.380 (0.289)	0.394 (0.272)	0.578 (0.295)	212.984 (43.325)
									
EtOH	30	0.315 (0.269)	0.201 (0.202)	4.588 (3.940)	3.562 (3.119)	0.478 (0.312)	0.229 (0.262)	0.575 (0.312)	220.103 (51.785)
									
F *P*		0.088 0.768	0.187 0.666	0.796 0.374	0.424 0.516	2.439 0.121	8.295 0.005	0.002 0.968	0.547 0.461

Abbreviations: BED, binge eating disorder; EtOH, alcohol dependent; HV, healthy volunteer; Meth, methamphetamine-dependent; OCD, obsessive-compulsive disorder.
