# Supplementary material for: Disorders of compulsivity: a common bias towards learning habits
Source: Mol Psychiatry. 2014 May 20;20(3):345–52. doi: 10.1038/mp.2014.44 (PMC4351889; doi:10.1038/mp.2014.44)
Supplement: Supplementary Information [file mp201444x1.doc]

**Supplementary information**

**Supplemental Methods**

*Recruitment*

Obese subjects (>30 Body Mass Index, BMI) with binge eating disorder (BED) and obese controls without BED (Obese), obsessive compulsive disorder (OCD) and abstinent alcohol dependent (EtOH) were recruited via community and university-based advertisements in the East Anglia region. Age- and gender-matched healthy volunteers (HV) (3:1 HV matching were used for the obese subjects with and without BED, OCD and EtOH subjects) were recruited via community and university-based advertisements in the East Anglia region. Abstinent methamphetamine dependent subjects (Meth) were also recruited from an inpatient rehabilitation center in Eden Prairie, Minnesota, USA. HV were recruited from community advertisements in Minneapolis. As this HV group were not matched for gender or IQ, additional HV were recruited in a 3:1 ratio who were matched for age, gender and IQ to the methamphetamine subjects from community advertisements in the East Anglia area. Primary diagnoses were confirmed by a psychiatrist (VV, JG or CR) using the Diagnostic and Statistical Manual of Mental Disorders, Version IV (DSM IV-TR) criteria for substance dependence or Research Diagnostic Criteria for BED .

Subjects >18 years old were included. HV, Obese subjects with or without BED, OCD and EtOH subjects were excluded if they had a current major depression or other major psychiatric disorder including substance addiction, major medical illness or were on psychotropic medications. OCD subjects were included if they had a YBOCS score >11. Subjects were screened for comorbid psychiatric disorders with the Mini International Neuropsychiatric Inventory. Subjects were excluded if they had positive urine drug screens or alcohol breathalyzer tests on the day of testing.

Meth subjects were tested one week to one year after abstinence and were excluded if they had current major depressive episode of moderate severity (Beck Depression Inventory >20), other major psychiatric history or major medical illness. Since the frequency of HIV is high, a sub-analysis was conducted to assess the role of HIV. Other forms of substance addiction were allowed assuming the primary drug for rehabilitation admission was methamphetamine (self-identified, highest frequency use and escalating use prior to admission). Regular drug screens were conducted at the rehabilitation centre. Etoh subjects were tested 2 weeks to 1 year after abstinence and >1 week after discontinuation of long-acting benzodiazepines used during detoxification. All psychiatric diagnoses were confirmed by a psychiatrist using DSM IV-TR criteria.

For HV, obese subjects with and without BED, OCD and EtOH, two separate specifically designed questionnaires were used to assess drug use (e.g. type, duration of use, amount per week, last use). Psychiatric disorders were screened using the Mini International Neuropsychiatric Interview . Subjects completed the UPPS-P Impulsive Behaviour Scale and Beck Depression Inventory . Further information included duration of methamphetamine use, duration of abstinence and Penn Craving Scale for Meth subjects, Binge Eating Scale (BES) for BED subjects, Yale Brown Obsessive Compulsive Scale for OCD subjects and the Alcohol Use Disorders Identification Test (AUDIT). The National Adult Reading Test was used to obtain an index of premorbid IQ.

Subjects were paid for their study participation time and told they could receive an additional amount (equivalent to £5) for their performance. Subjects in the study in Minnesota were given the equivalent amount in a department store gift card.

*Image acquisition*

Imaging data was acquired using a Siemens 3T Tim Trio 3T scanner (Siemens Medical System Systems, Erlangen, Germany) at the Wolfson Brain Imaging Centre at the Uiversity of Cambridge. For the study with young healthy volunteers only, data was acquired with a 12 channel head coil. For the study with Obese subjects and healthy volunteers, data was acquired with a 32 channel head coil. The data sets from acquisition using differing head coils were analyzed separately. Anatomical images were obtained using a T1-weighted structural image using an MPRAGE sequence (TR=2300 ms; TE=2.98 ms; FOV 240 x 256 x 176 mm, voxel size 1x1x1 mm).

Imaging data were preprocessed using Statistical Parametric Mapping software (SPM8) (<http://www.fil.ion.ucl.ac.uk/spm>). The 3D T1-weighted images were reoriented with the origin set close to the anterior commissure. The images were then segmented into different tissue classes in New Segment which uses tissue probability maps to assign a probability of each voxel belonging to a particular tissue class. The volume of the grey matter, white matter and CSF tissue classes were summed to provide an estimate of total intracranial volume. The grey matter images were used to generate a custom template using DARTEL , a diffeomorphic method, which iteratively defines the parameters used to warp each subject native grey matter image to a common space. To transform these template-space images into ICBM152 MNI space, the DARTEL template was registered to the tissue probability maps using an affine transformation incorporated into the warping process. Images were smoothed using an 8 mm FWHM isotropic Gaussian kernel in the final normalization step.

Grey matter volume in healthy volunteers was analyzed using a general linear model in a regression analysis with *w* as a dependent factor. Grey matter volume in Obese subjects with and without BED were compared with healthy volunteers using independent t-tests. A regression analysis was conducted with the regressors of BES or Body Mass Index. Obese subjects with BED were compared with those without BED using a t-test with gender as a covariate of no interest. Obese subjects with BED were compared with matched healthy volunteers and obese subjects without BED were compared with matched healthy volunteers using t-tests.

In all analyses, total intracranial volume using proportional scaling with an explicit mask using the brain mask template was used. The ventral striatal anatomical region of interest (ROI), previously used in other studies , had been hand drawn in MRIcro following the definition of ventral striatum by Martinez et al. . The bilateral caudate and putamen, orbitofrontal cortex (Brodmann Area 11), lateral prefrontal cortex (Brodmann Area 9 and 46) and parietal cortex (superior and inferior parietal cortex) ROIs were obtained from aal templates in WFUPickAtlas SPM Toolbox . In the comparison of BED and Obese subjects, the analysis was also conducted with age and BDI scores as covariates of no interest. All clusters greater than 5 voxels for FWE whole brain corrected P<0.05 were considered significant. For hypothesized regions, small volume correction (SVC) FWE whole brain corrected p<0.05 was considered significant.

*Behavioural outcomes*

In the computational model, parameter values are determined by integrating effects associated with sequences of many choices. A more direct, though less powerful, way of assessing group differences is to examine pairs of successive choices, studying how any tendency of subjects to Stay with the same Stage 1 choice or Switch following Outcome (Reward or No Reward) depends on the Frequency of the Stage 1 to Stage 2 transition (Common (P=0.70) or Rare (P=0.30)). Under the habitual system, a Stage 1 choice would be more likely to be repeated (Stay) when followed by Reward, regardless of whether the transition was Common or Rare. Thus a habitual strategy would reflect a main effect of Outcome in Stay probability (Table S3 and Figure S1). Conversely, a goal-directed strategy would tend to Switch its subsequent Stage 1 choice if it was Rewarded but the transition was Rare. Given knowledge of the task structure, the *other* choice at Stage 1 would more likely lead to the rewarded Stage 2 choice. Thus, a goal-directed strategy would reflect an interaction between Outcome x Frequency.

We used a mixed effects logistic regression for the Stay probability analysis with Outcome (Reward or No Reward), Frequency (Rare or Common), and Group as factors, comparing all subject groups and healthy volunteers. Outcome, frequency, their interaction, and the intercept were taken as random effects, i.e. varying across subjects. We estimated the regression coefficients using the lme4 linear mixed effects package in the R statistical environment.

**Supplemental Data**

*Subject characteristics*

Thirty-one obese BED and 31 obese controls were recruited from the East Anglia area and compared to age- and gender-matched HV (N=93 and N=93 respectively) (Table S1). Thirty-three OCD subjects (YBOCS score 21.34 (SD 5.47)) were recruited and compared to age- and gender-matched HV (N=99 from the East Anglia area) (Table S2). One OCD subject with high depression scores was excluded. Twenty-two OCD subjects were on antidepressants. Twenty-three Meth subjects (reported in mean (SD): days abstinent: 79.16 (140.28); years ever used: 10.16 (6.31); years of heavy use: 2.60 (2.51); Penn Craving Scale: 15.17 (9.17)) (Table S2). In the Meth subjects, all subjects had completed high school and 18/23 had some college, graduate or post-graduate education. Data from 1 Meth subject were excluded due to a moderately severe current major depressive episode. Six Meth subjects had a concurrent alcohol use disorder and 21 used nicotine daily. Meth subjects had the following comorbid psychiatric diagnoses (lifetime major depression 4; panic disorder 1; post-traumatic stress disorder 1; obsessive compulsive disorder 1; anorexia nervosa/bulimia 1; compulsive sexual behaviors 2; attention deficit hyperactivity disorder 4). Meth subjects were on the following medications (antidepressants 9; mood stabilizer 3 (used also for pain); neuroleptic 2; medication status unknown 2). Twenty HV were recruited from the community in Minnesota. An additional forty-six additional HV matched for age, gender and IQ from community settings in East Anglia were also recruited. Thirty EtOh subjects (AUDIT 19.58 (SD 14.10); weeks abstinent 16.17 (SD 16.07); years of dependence 12.85 (SD 8.26); Units/day 27.86 (SD 14.18)) were compared with 90 age- and gender-matched healthy volunteers. EtOH subjects were on the following medications (acamprosate 2; disulfiram 1).

For the VBM study focusing on HV, 33 HV (mean age 23.22 (SD 2.75); 19 males) underwent scanning. For the VBM Study focusing on Obese subjects, 20 Obese subjects with BED (male gender N=8; age in years 43.95 (SD 9.47); BMI 34.12 (SD 5.49); BES 24.70 (SD 7.57)) were compared with 20 Obese subjects without BED (male gender N=11; age in years 44.70 (SD 10.12), t=0.24, p=0.81; BMI 32.88 (SD 3.53), t=0.85, p=0.40; BES 8.67 (SD 7.21), t=6.86, p<0.001)).

*Model parameters*

In the Meth subjects, the HIV status of 2 subjects was not known. There were no differences in *w* in Meth subjects between HIV + versus HIV –(N=11, w=0.199 (SD 0.190); N=11, w=0.243 (SD 0.289) respectively, p=0.679), high versus low or no nicotine use (high: N=10, w=0.228 (SD 0.261); low: N=10 w=0.245 (SD 0.205), p=0.870), with or without ADHD (N=7, w=0.146 (SD 0.190); N=17, w=0.258 (SD 0.250) respectively, p=0.303). In the Meth group, there was no correlation between model parameters and duration of Meth use, duration of abstinence or Penn Craving Scale (P>0.05). In the OCD group, there were no differences in model parameters between those on antidepressants and not on medications (P>0.05). There was no correlation between model parameters with YBOCS (P>0.05).

In healthy volunteers, there was a positive correlation between the perseveration index and UPPS (r=-0.21, p=0.04) related to positive urgency (r=-0.24, p=0.02). There were no correlations between the perseveration index or w with age, IQ or Beck Depression Inventory scores (p>0.05). There were no gender differences in *w* or perseveration index scores (p>0.05).

*Behavioural outcomes*

Figure S1 depicts the frequency of switching as a function of the previous trial’s events, in HV and the other subject groups, respectively. The pattern of results appears to show both a main effect of Outcome and its interaction with Transition in HV (and also in the Obese group), indicating a mixture of model-based and model-free strategies and consistent with previous data using this task. All groups with disorders of compulsivity, however, appear to show only a main effect of Outcome, the marker of model-free learning only.

To quantify these impressions and compare groups directly on these measures, we conducted a mixed effects logistic regression of stay or switch choices. In the logistic regression of behavioural outcomes focusing on HV alone, there was indeed a main effect of Transition (Z=-2.66, p=0.008), Outcome (Z=9.43, p<2e-16) and of Transition x Outcome (Z=-5.72, 1.05e-8) indicating parallel use of both goal-directed and habitual processes.

In the comparison of subject groups and HV, there was a main effect of Outcome (P<2e-16) in Stay probability indicating that subjects across all groups were more likely to Stay after reward (Table S3 and Figure S1). In the critical Outcome x Frequency interaction, compared to HV, there were interactions for BED (p=0.045) and OCD (p=0.04) with a trend in Meth (p=0.07) suggesting less use of goal-directed processes compared to HV. There was no significant Outcome x Frequency interaction in the Obese group (p=0.56). Altogether, these results are consistent with those from the computational model fits demonstrating greater relative engagement of habits than goal-directed control in groups BED, OCD with a trend in Meth groups but not in Obese subjects without BED.

**Supplementary References**

1. Association AP. Diagnostic and statistical manual of mental disorders (4th Ed., text rev). Washington, D.C.: American Psychiatric Association; 2000.

2. Sheehan DV, Lecrubier Y, Sheehan KH, Amorim P, Janavs J, Weiller E, et al. The Mini-International Neuropsychiatric Interview (M.I.N.I.): the development and validation of a structured diagnostic psychiatric interview for DSM-IV and ICD-10. J Clin Psychiatry. 1998;59 Suppl 20:22-33;quiz 4-57.

3. Whiteside SP, Lynam DR. The five factor model and impulsivity: using a structural model of personality to understand impulsivity. Personality and Individual Differences. 2001;30(4):669-89.

4. Beck AT, Ward CH, Mendelson M, Mock J, Erbaugh J. An inventory for measuring depression. Archives of general psychiatry. 1961;4:561-71.

5. Gormally J, Black S, Daston S, Rardin D. The assessment of binge eating severity among obese persons. Addictive behaviors. 1982;7(1):47-55.

6. Nelson HE. National Adult Reading Test. Windosr, UK: NFER-Nelson; 1982.

7. Ashburner J. A fast diffeomorphic image registration algorithm. NeuroImage. 2007;38(1):95-113.

8. Murray GK, Corlett PR, Clark L, Pessiglione M, Blackwell AD, Honey G, et al. Substantia nigra/ventral tegmental reward prediction error disruption in psychosis. Molecular psychiatry. 2008;13(3):239, 67-76.

9. Martinez D, Slifstein M, Broft A, Mawlawi O, Hwang DR, Huang Y, et al. Imaging human mesolimbic dopamine transmission with positron emission tomography. Part II: amphetamine-induced dopamine release in the functional subdivisions of the striatum. Journal of cerebral blood flow and metabolism : official journal of the International Society of Cerebral Blood Flow and Metabolism. 2003;23(3):285-300.

10. Maldjian JA, Laurienti PJ, Kraft RA, Burdette JH. An automated method for neuroanatomic and cytoarchitectonic atlas-based interrogation of fMRI data sets. NeuroImage. 2003;19(3):1233-9.

**Legend**

Figure S1. Stay probability:

A.Simulated Stay probabilities (likelihood of staying with same choice in stage 1) shown for habit and goal-directed strategy use. B. Logistic regression of Stay probabilities in healthy volunteers. C. Logistic regression of subject groups. Significant findings are presented in the text and Table S3. Abbreviations: BED = Obese with binge eating disorder; Obese = Obese without BED; Meth = methamphetamine-dependence; OCD = obsessive compulsive disorder

Table S1. Subject characteristics of Obese and alcohol dependent subjects

Data are reported in mean (SD).

Abbreviations: HV=healthy volunteers; BED=binge eating disorder; EtOH=abstinent alcohol dependent subjects; N=number of subjects; SD=standard deviation; BDI=Beck Depression Inventory; UPPS= UPPS Impulsive Behaviour Scale; BMI=Body Mass Index; BES=Binge Eating Scale

Table S2. Subject characteristics of OCD and Methamphetamine dependent subjects

Data are reported in mean (SD).

Abbreviations: HV=healthy volunteers; OCD=obsessive compulsive disorder; Meth=Methamphetamine dependent; Minn=Minnesota; N=number of subjects; BDI=Beck Depression Inventory; UPPS=UPPS Impulsive Behaviour Scale

Table S3. Stay probability

* represents significant findings

Abbreviations: HV=healthy volunteers, BED=binge eating disorder, Meth=methamphetamine-dependent, OCD=obsessive-compulsive disorder

**Table S1. Subject characteristics of Obese and Alcohol dependent subjects**

|  | HV BED | BED | P | HV Obese | Obese | P | HV EtOH | EtOH | P |
| --- | --- | --- | --- | --- | --- | --- | --- | --- | --- |
| N | 93 | 31 |  | 93 | 31 |  | 90 | 30 |  |
| Age in years | 42.72 (10.36) | 42.79 (9.02) | 0.03  0.97 | 43.15 (10.22) | 44.24 (9.39) | 0.53 0.59 | 42.52 (9.15) | 41.40 (11.57) | 0.54  0.59 |
| Males N | 36 | 12 |  | 57 | 19 |  | 54 | 18 |  |
| IQ | 114.18 (5.70) | 115.11 (6.72) | 0.75  0.45 | 115.38  (5.91) | 114.96 (6.38) | 0.34 0.74 | 114.88 (6.41) | 114.32 (6.76) | 0.41 0.68 |
| BDI | 5.03 (4.77) | 12.14 (6.55) | 6.51  <0.001 | 5.31 (4.68) | 7.95 (6.10) | 1.56 0.12 | 5.14 (4.66) | 12.89 (9.29) | 0.44 0.66 |
| UPPS | 123.29 (18.58) | 133.18 (19.36) | 2.54  0.01 | 121.82 (22.67) | 128.59 (21.32) | 1.25 0.22 | 122.57 (22.58) | 154.25 (20.14) | 6.83 <0.001 |
| BMI | 23.96 (2.92) | 34.97 (5.56) | 14.17 <0.001 | 23.54 (2.89) | 31.49 (3.60) | 12.45 <0.001 | 23.59 (3.01) | 22.94 (2.87) | 1.04 0.30 |
| BES | 6.97 (6.55) | 25.03 (7.47) | 12.82 <0.001 | 6.57 (6.13) | 8.86 (7.25) | 1.72 0.09 | 6.43 (6.18) | 7.69 (10.31) | 0.81 0.42 |

Table S2. Subject characteristics of OCD and Methamphetamine dependent subjects

|  | HV OCD | OCD | P | HV Meth Minn | HV Meth total | Meth | P |
| --- | --- | --- | --- | --- | --- | --- | --- |
| N | 96 | 32 |  | 20 | 66 | 22 |  |
| Age in years | 36.93 (9.08) | 35.68 (12.57) | 0.61 0.54 | 33.50  (7.78) | 32.19 (7.92) | 31.05 (4.78) | 0.64 0.53 |
| Males N | 45 | 15 |  | 16 | 63 | 21 |  |
| IQ | 115.11 (5.91) | 114.68 (6.29) | 0.35 0.73 | 112.01 (4.71) | 111.97 (5.98) | 108.89 (4.59) | 2.21 0.03 |
| BDI | 5.17 (4.19) | 15.09 (7.33) | 9.45  <0.001 | 5.35 (5.33) | 5.71 (5.64) | 15.32 (8.13) | 6.17 <0.001 |
| UPPS | 123.65 (20.70) | 119.34 (18.23) | 1.05 0.30 | 120.94 (21.27) | 122.91 (22.35) | 156.71 (22.47) | 6.13 <0.001 |

**Table S3. Stay probability**

|  | BED  Z  P | Obese  Z  P | OCD  Z  P | Meth  Z  P | Z  P |
| --- | --- | --- | --- | --- | --- |
| Intercept |  |  |  |  | 1.22  < 2e-16 |
| Outcome |  |  |  |  | 9.61  <2e-16* |
| Frequency |  |  |  |  | -2.57  0.01* |
| Group (vs HV) | 0.15  0.88 | 0.64  0.52 | 0.001  0.99 | -0.69  0.49 |  |
| Frequency x Outcome |  |  |  |  | -5.91  3.53e-9* |
| Frequency x Group | 1.44  0.15 | 0.51  0.61 | 0.95  0.34 | 0.37  0.71 |  |
| Outcome x Group | -0.36  0.72 | 0.34  0.74 | 0.64  0.52 | -1.23  0.22 |  |
| Frequency x Outcome x Group | 2.00  0.045* | 0.58  0.56 | 2.91  0.004* | 1.82  0.07 |  |
