# Supplementary figures and images for: Disorders of compulsivity: a common bias towards learning habits
Source: Mol Psychiatry. 2014 May 20;20(3):345–52. doi: 10.1038/mp.2014.44 (PMC4351889; doi:10.1038/mp.2014.44)

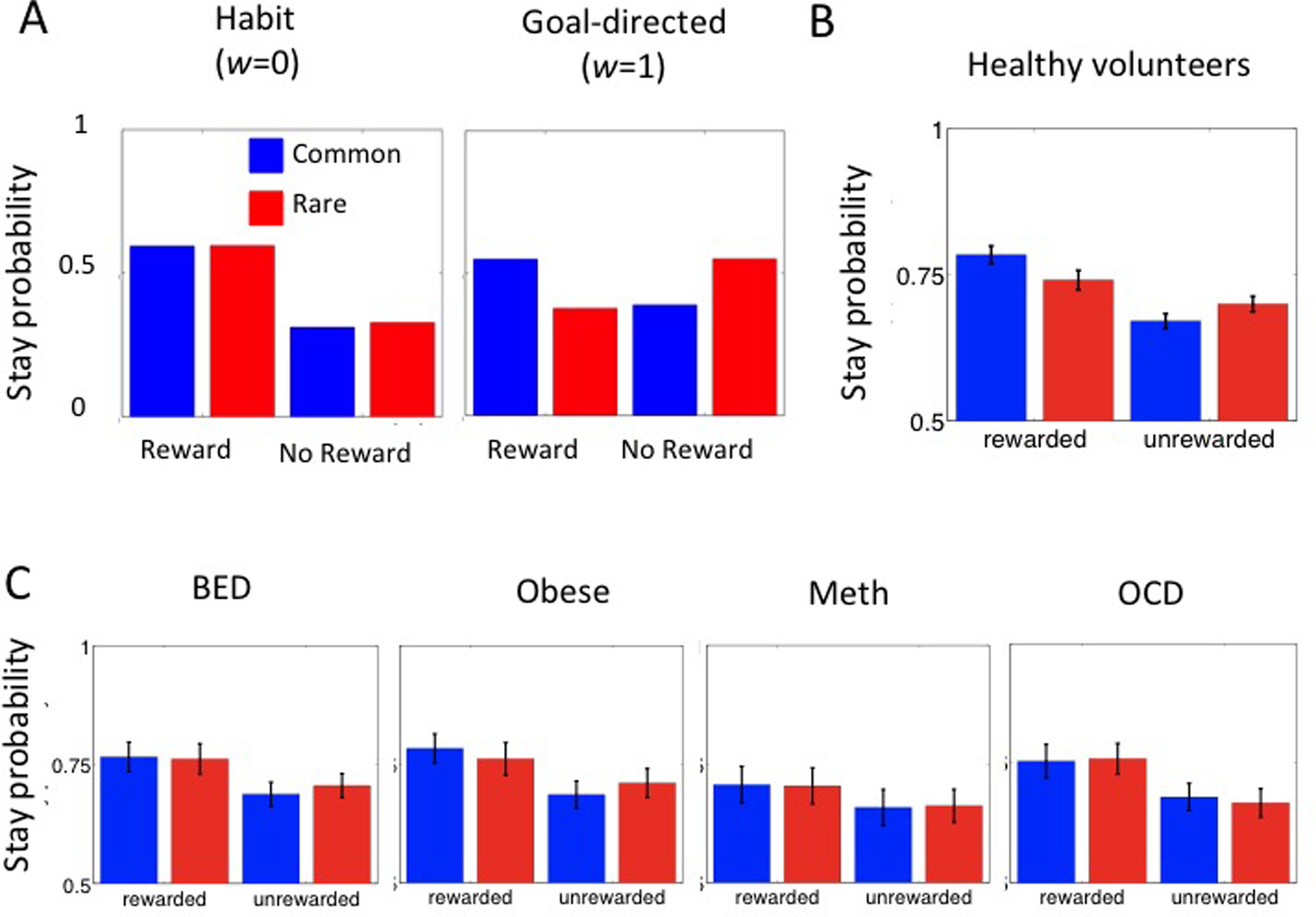

Supplement: Supplementary Figure [file mp201444x2.tif]
